# Local cryptic diversity in salinity adaptation mechanisms in the wild outcrossing *Brassica fruticulosa*

**DOI:** 10.1073/pnas.2407821121

**Published:** 2024-09-24

**Authors:** Silvia Busoms, Ana C. da Silva, Glòria Escolà, Raziyeh Abdilzadeh, Emma Curran, Anita Bollmann-Giolai, Sian Bray, Michael Wilson, Charlotte Poschenrieder, Levi Yant

**Affiliations:** ^a^Department of Plant Physiology, Universitat Autònoma de Barcelona, Barcelona 08193, Spain; ^b^School of Life Sciences, Faculty of Medicine & Health Sciences, University of Nottingham, Nottingham NG7 2RD, United Kingdom; ^c^Department of Cell and Developmental Biology, John Innes Centre, Norwich Research Park, Norwich NR4 7UH, United Kingdom; ^d^School of Computer Sciences, Faculty of Science, University of Nottingham, Nottingham NG7 2RD, United Kingdom; ^e^Department of Botany, Faculty of Science, Charles University, Prague 128 01, Czech Republic

**Keywords:** adaptation, salinity, evolution, population genomics, Brassicaceae

## Abstract

It is normally supposed that populations of the same species should evolve shared mechanisms of adaptation to common stressors due to evolutionary constraint. Here, we describe a system of within-species local adaptation to coastal habitats, *Brassica fruticulosa,* and detail surprising strategic variability in adaptive responses to high salinity. These different adaptive responses in neighboring populations are evidenced by transcriptomes, diverse physiological outputs, and distinct genomic selective landscapes. In response to high salinity Northern Catalonian populations restrict root-to-shoot Na^+^ transport, favoring K^+^ uptake. Contrastingly, Central Catalonian populations accumulate Na^+^ in leaves and compensate for the osmotic imbalance with compatible solutes such as proline. Despite contrasting responses, both metapopulations were salinity tolerant relative to all inland accessions. To characterize the genomic basis of these divergent adaptive strategies in an otherwise non-saline-tolerant species, we generate a long-read-based genome and population sequencing of 18 populations (nine inland, nine coastal) across the *B. fruticulosa* species range. Results of genomic and transcriptomic approaches support the physiological observations of distinct underlying mechanisms of adaptation to high salinity and reveal potential genetic targets of these two very recently evolved salinity adaptations. We therefore provide a model of within-species salinity adaptation and reveal cryptic variation in neighboring plant populations in the mechanisms of adaptation to an important natural stressor highly relevant to agriculture.

## Significance

One might expect that closely related populations of a given species should adapt to the same environmental stressor in the same way due to genetic or physiological constraints. However, this is not commonly tested due to practical limitations. Here, we show that, even at the level of neighboring populations, contrasting adaptive strategies control adaptive responses to high coastal salinity in *Brassica fruticulosa*, a close wild relative of many crops of worldwide importance. This indicates multiple options for engineering an agriculturally crucial adaptation: soil salinization. These results will be of interest to not only those studying fundamental mechanisms of adaptation, but also resilience improvement in Brassica species.

Today’s accumulation of high-profile cases detailing repeated evolution capture the fascination of biologists. Independently evolved adaptive coloring shifts in mammals and insects, defensive armor in fish, and serpentine and altitude adaptation in plants: these all present not only additional evidence for candidate mechanisms underlying adaptations, but also an optimistic outlook toward “predicting” the course of evolution and inspiring expositions for the public (1–6). Given these iconic cases, an expectation may arise that even at the functional level, neighboring populations of the same species should, due to genetic or developmental constraints and mutation limitation, share evolved strategies of adaptation to the same stressors (7). The logical extension is that natural selection might be expected to predictably drive the origin and maintenance of adaptations at strategic or mechanistic levels. However, this idea has not been sufficiently tested due to restraints on study systems, sampling, resolution, and scale (8). We thus lack a clear understanding of how often an expectation of uniform or repeatable species-wide adaptation strategies is violated in favor of diversity even within single species.

Here, we test this expectation by taking a “hyperlocal” approach in the study of plant adaptation to coastal stressors, focusing on adaptation to high coastal salinity in a strip of coastline in Catalunya, Northern Spain. Previous work on local adaptation of *Arabidopsis thaliana* in this region detailed geographically and temporally fine-scale adaptive variation in fitness-related traits across environmental salinity gradients, even at the scale of a few kilometers (9, 10). This region is characterized by a positive gradient of soil salinity from inland to the coast, shaping plant species communities and driving the evolution of salinity tolerance mechanisms at the local population- (deme-) level (11). Plant evolutionary responses to these conditions have been observed even in the selfer *A. thaliana* at fine (3 to 5 km) scale, resulting in functionally adaptive variation (12). Functional confirmation of this is evidenced by selective sweep of a hypomorphic ion transporter HKT1;1, which modulates Na^+^ leaf concentrations in response to rapid (monthly) temporal and spatial variation in rainfall and soil salinity (9).

Unfortunately, work in *A. thaliana* has two major limitations: first, due to its overwhelmingly selfing reproductive mode, relative to its outcrossing relatives *A. thaliana* has 10-fold lower genetic diversity and high rates of spontaneous, population-specific mutations (13). This low diversity also has important consequences in respect to increased homozygosity and effective population size, resulting in genetic drift, reduced effective recombination rates, genomic background effects, and the fixation of maladaptive alleles (reviewed in ref. 14). Second, *Arabidopsis* is substantially divergent from important Brassica crops, limiting the translational potential of discoveries in this otherwise convenient lab model. Wild outcrossing Brassicas, on the other hand, harbor higher levels of genetic diversity, directly facilitating studies of adaptation (15). Motivated by these considerations, we searched for wild Brassicaceae species with contrasting, recently evolved (within-species) phenotypes in complex coastal adaptations, focusing specifically on salinity tolerance. This resulted here in the identification of a model for local adaptation to coastal salinity, *Brassica fruticulosa*, and allows us to test hypotheses regarding the scale of local adaptation to high coastal salinity.

The genus *Brassica* belongs to the Brassicaceae (mustard) family and contains nearly 100 species, many of which are grown globally as vegetables like cabbage, broccoli, kale, and radish, as mustards, as oil crops (placing 3rd after palm and soy), and as fodder for animal feed (16). Brassicas are widely proficient at adapting to new habitats due to recent and recurrent polyploidy events, hybridization, and plastic genomes. These characteristics also make them great targets for genetic manipulation to further enhance resilience (17).

Here, we first perform a large-scale, genus-wide natural variation survey of diverse, wild outcrossing Brassicas in coastal Northeast Spain, eventually testing six candidate species for within-species adaptation to high salinity. From these, we identify and develop one particularly promising model of within-species variation in adaptation to extreme salinity and complex coastal stressors, *B. fruticulosa.* First described in 1792 by Cirillo (18), *B. fruticulosa* has not yet been recognized as harboring population-specific salinity adaptation. This has been a missed opportunity, as *B. fruticulosa* is closely related to *Brassica rapa* (19, 20) and shares many affinities with this global crop. We then assemble the *B. fruticulosa* genome using Oxford Nanopore long read sequencing polished with Illumina short reads, and sequence 90 individuals from 18 populations (nine coastal, nine inland) contrasting in salinity and soil parameters defined by ionome levels in leaves and soil in the root space of every individually sequenced wild plant. Using transcriptome data of leaves and roots, we reveal divergent adaptive strategies in response to high salinity in neighboring plant populations. We then perform common garden, physiological, and ion homeostasis experiments to detail these different strategies that evolved in closely neighboring adapted plant populations. Finally, we perform environmental association analysis (EAA) (with soil ionome as phenotype) and genome scans by ecotype to seek a genomic basis of divergent adaptative strategies to high salinity in neighboring *B. fruticulosa* populations. Taken together, these experiments reveal contrasting adaptive responses to extreme salinity, at the local scale, differing mechanistically at the scale of kilometers.

## Results and Discussion

### A Region-Exhaustive Screen for Fine-Scale Salinity Adaptation in Catalonia.

Situated in Northeast Spain, Catalonia harbors a diverse flora ranging across a positive gradient of soil salinity from inland to the coast. This salinity gradient acts as a selective agent upon coastal plants to develop mechanisms of adaptation to high coastal salinity (11) and is accompanied by other local stressors (such as impoverished soils, alkalinity, or drought), which are understood in good resolution (21–23). These selective agents, combined with the fact that the Iberian region has served as an important glacial refugium for many species, have resulted in dramatic local plant diversification. For example, in the most well-studied system in terms of genome resources, African and Iberian *A. thaliana* harbors the largest genomic diversity of the entire species distribution (24) and has shown significant adaptive variation in fitness-related traits across environmental gradients (e.g., refs. 25 and 26). As members of the same family, Brassica species benefit from the extensive molecular genetics and genomic tools developed for Arabidopsis. The close relationship among Brassica species, along with the abundance of wild relatives and minor crop species in the broader Brassicaceae tribe, makes it a valuable model for studying genes of interest for interspecific hybridization and crop improvement (17).

**Fig. 1.**
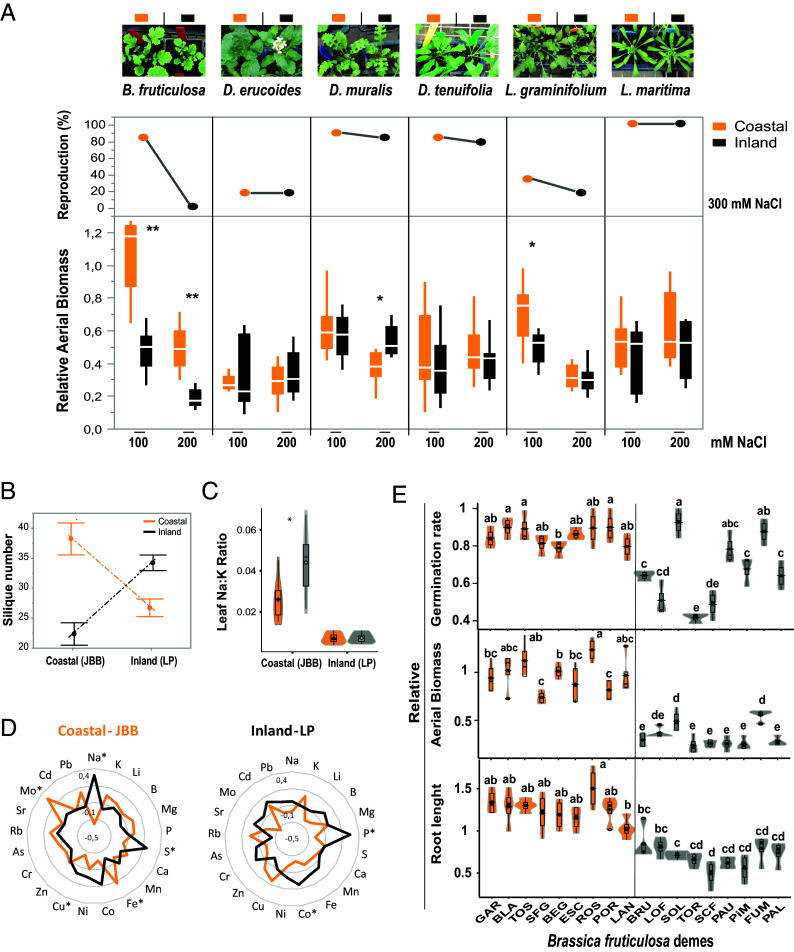
*B. fruticulosa* exhibits marked within-species variation in performance under salinity relative to other local Brassicaceae species in a region-exhaustive survey. (*A*) Relative aerial biomass (DW Treatment/DW Control) of *B. fruticulosa*, *D. erucoides, D. muralis*, *D. tenuifolia, L. graminifolium*, and *L. maritima* plants from coastal (orange) and inland (black) populations cultivated in common soil irrigated with 100 mM NaCl or with 200 mM NaCl for 1 wk. Asterisks indicate significant differences (ANOVA, *P < 0.05, **P < 0.01) in aerial biomass between origin under these saline conditions. Percentage of plants able to produce seeds after exposure to 300 mM NaCl until maturation. Pictures representative of coastal and inland 21-d-old plants treated with 100 mM NaCl are above. (*B*) Fitness (silique number as proxy), (*C*) leaf Na:K ratio, and (*D*) leaf ionome profiles of *B. fruticulosa* plants from coastal (orange) and inland (black) populations cultivated in a coastal field common garden (JBB) and an inland field common garden (LP). (*E*) Relative (Treatment/Control) germination rate, aerial biomass, and root length of *B. fruticulosa* populations sown in MS plates (0 or 75 mM NaCl). Seedlings germinated without NaCl were transferred to hydroponic conditions and treated with 0 mM (Control) or 150 mM NaCl (Treatment) for 10 d. Letters indicate significant differences between populations (Tukey’s Honestly Significant Difference (HSD) test).

We thus performed a search for all Brassicaceae wild species in the region with contrasting interspecies phenotypes in salinity tolerance. We first identified populations from 13 wild Brassica species and georeferenced these from available databases (*Materials and Methods*). Six of these 13 species were found in more than two coastal and two inland sites. Seeds from these species (*B. fruticulosa*, *Lepidium graminifolium*, *Lobularia maritima*, *Diplotaxis tenuifolia, Diplotaxis erucoides,* and *Diplotaxis muralis*) were collected (Dataset S1) and progeny were tested for salinity tolerance. Salinity tolerance in *L. maritima*, *D. tenuifolia,* and *D. muralis* was uniformly high in both coastal and inland ecotypes, with plants completing their life cycle under exposure to 300 mM of NaCl regardless of their coastal or inland origin (Fig. 1*A*). Coastal and inland *D. erucoides* and *L. graminifolium* plants exhibited equally higher sensitivity, while still enduring 200 mM NaCl for 2 to 3 wk but not reproducing after longer exposures. Of all the Brassicaceae species recorded in the region and identified both inland and coastally, only *B. fruticulosa* exhibited a within-species contrast in the ability to grow and reproduce in high soil salinity (Fig. 1*A* and Dataset S1).

### Local Adaptation and Intraspecific Variation for Salinity Tolerance in *B. fruticulosa*.

Having found marked salinity tolerance of particular *B. fruticulosa* populations under controlled laboratory conditions, we tested for local adaption to inland and/or coastal sites. We performed a reciprocal transplant experiment in two representative local field sites (JBB-coastal and LP-inland), which had been established for similarly testing local adaptation in wild *A. thaliana* (11, 26). As a proxy for the fitness of each plant, we counted the number of siliques produced. Additionally, 30 d after germination one leaf per plant was harvested for ionomic analysis. In across-site comparisons, both coastal and inland plants performed better in their home environment (Fig. 1*B*). In within-site comparisons, coastal plants outperformed inland plants when both were grown together in the coastal environment, whereas inland plants displayed a significant fitness advantage compared with coastal plants when both were grown inland (Fig. 1*B*), indicating that both the coastal and inland populations are locally adapted.

Having established this local adaptation of coastal and inland populations, and in particular, the relative salinity tolerance of coastal populations (Fig. 1*A*), we then focused on possible strategies underlying the salinity tolerance of the coastal populations. This tolerance may be caused by either Na^+^ exclusion or the accumulation of Na^+^ and internal tissue tolerance mechanisms (12, 27). Whole leaf ionome analysis revealed that *B. fruticulosa* plants from coastal and inland populations did not differ in their accumulation of Na^+^ or K^+^ under inland conditions. However, in coastal common gardens, inland plants accumulated more Na^+^ than coastal plants, resulting in a significant increase in the Na:K ratio (Fig. 1 *C* and *D* and Dataset S3). These results indicate that coastal populations restrict leaf Na^+^ accumulation, maintaining a significantly lower Na:K ratio under coastal conditions. Conversely, at the inland site, some essential nutrients such as *P* were higher in the *B. fruticulosa* inland populations (Fig. 1*D*), reflecting the better growth and nutritional status of inland plants in their local origin.

To explicitly test for intraspecific salinity tolerance of all *B. fruticulosa* populations, a hydroponic experiment was conducted to ensure uniform NaCl treatment. Thirty seeds from each population were placed in square plates with ½ MS medium (pH 6) or ½ MS medium with 75 mM of NaCl (pH 6). One-week-old seedlings from the control plates were transferred to a hydroponic system filled with 0.5-strength Hoagland solution (pH 6). After a week, salinity was gradually increased (until a maximum of 150 mM NaCl, pH 6) in the group of treated plants. More than 75% of seeds from coastal populations germinated under saline conditions. Contrastingly, 6 of the 9 inland populations had a relative germination rate below 70% (Fig. 1*E*). Some inland populations (SOL, PAU, and FUM) could germinate well in 75 mM NaCl, but already at the seedling stage they suffered a significant reduction of growth and root development when exposed to salinity (150 mM of NaCl; Fig. 1*E*). All *B. fruticulosa* coastal populations outperformed the inland populations in terms of aerial biomass (Fig. 1*E*), explicitly identifying salinity tolerance in these populations (Dataset S4).

### Long Read-Based *B. fruticulosa* Genome Assembly and Annotation.

As an initial step in determining the genomic basis of the observed variation in salinity tolerance in *B. fruticulosa*, we generated a reference genome of an individual from a particularly salinity-tolerant population, TOS (Tossa de Mar, Catalonia, Spain), using long-read Oxford Nanopore Technology (ONT). The alignment and accuracy corrections of reads resulted in a final purged assembly of 351 mb with an N50 of 470 kb. Given a repetitive ratio of 0.56 (312 mb), we expect this assembly to be complete for the nonrepetitive gene space. This was confirmed through benchmarking analysis using BUSCO (28) against brassicales_odb10. This showed high completeness (97.1% complete BUSCOs) (*SI Appendix*, Fig. S1). Finally, gene models were predicted by an evidence-guided annotation approach incorporating RNA-Sequencing (RNA-seq) data and cross-species protein alignments in BRAKER2 (29).

### Population Sequencing and Genetic Structure Analysis.

**Fig. 2.**
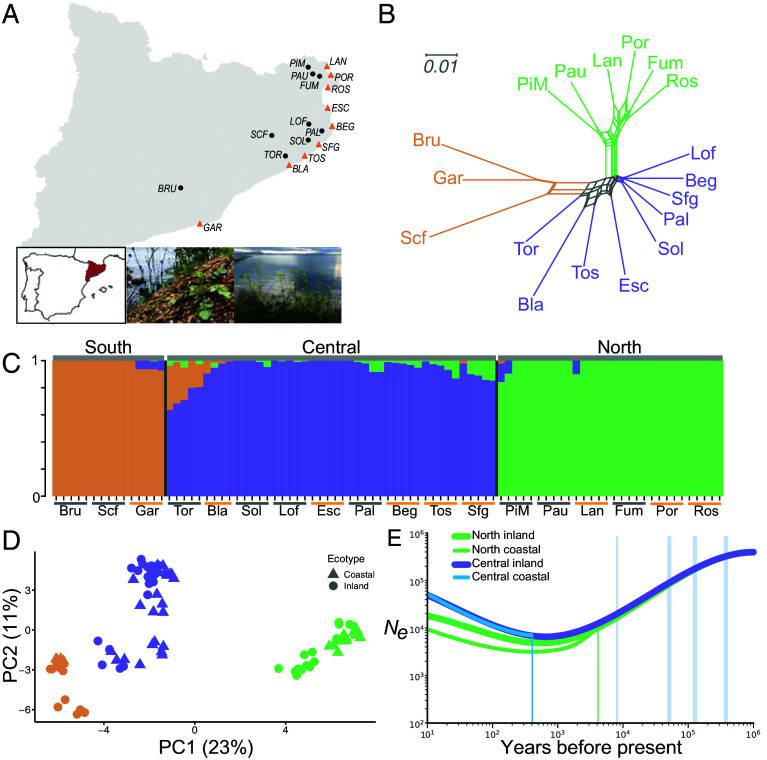
Population resequencing, genetic structure, and effective population size of *B. fruticulosa*. (*A*) Sampling map of Catalonia, Northern Spain, of the 18 populations sequenced. (*Inset*) Image of saline-tolerant coastal *B. fruticulosa.* (*B*) Nei’s genetic distances between populations visualized in SplitsTree. (*C*) fastSTRUCTURE analysis (k = 3, min MAF 2.5%) of the 89 individuals analyzed, with clusters corresponding tightly to geographic origin, but not to salinity tolerance or coastal/inland status. (*D*) PCA of all individuals. Coastal populations are represented by triangles and inland populations by circles. Northern populations are colored in green, Central in purple, and Southern in orange. (*E*) Effective population size (*Ne*) over time of focal metapopulations predicted by SMC++, along with split times (vertical blue and green lines). Historic relative cooling periods in this Mediterranean refugium are highlighted in light cyan.

To assess population- and individual-level demographic and selective landscapes, we sampled 18 populations (Fig. 2*A*) for genome sequencing across the reported range of *B. fruticulosa*. We Illumina sequenced a total of 90 individuals, selecting five individuals from each of the 18 populations (average depth per individual = 13.2×; Dataset S5). One sample, Bla5, was excluded due to suspicion of sample contamination.

To assess genetic structure, we performed fastSTRUCTURE (30) and NGSadmix (31) (Fig. 2*C*) on our 89 individuals (30,272 Linkage Disequilibrium (LD)-pruned, biallelic fourfold-degenerate Single Nucleotide Polymorphisms (SNPs); max 20% missing data; min Minor Allele Frequency (MAF) = 2.5%). K = 3 maximized marginal likelihood, consistent also with Discriminant Analysis of Principal Components analysis of the Bayesian Information Criterion in *adgenet* (32). The K = 3 result reflects a Northern group (including three coastal and three inland populations), a Central group (including five coastal and four inland populations), and a very small Southern group (with only two inland and one coastal population) that was excluded in the following analysis. Except for SCF, this grouping partitioned all samples by geographic region of origin (Fig. 2) and not by salinity tolerance status or local soil characteristics (Fig. 2*C*). Both Nei’s simple genetic distances visualized in SplitsTree (Fig. 2*B*) and Principal component analysis (PCA) (Fig. 2*D*) confirmed that geographic origin completely dominates over saline/nonsaline ecotype.

Consistent with neutral Tajima’s D for all populations (Dataset S5), we confirmed no evidence of drift or bottleneck by applying an extension of the Sequentially Markovian Coalescent [SMC++ (33)]. This approach uses the Site Frequency Spectrum (SFS), haplotype information, recombination, and LD patterns, targeted toward very recent demographic history and assessment of historical population sizes and splits. For the four metapopulations that form the focus of our analyses below (North and Central, coastal, and inland each), historical effective population sizes were stable or gradually expanding over the last 1,000 generations, with a gradual decline in deeper time, starting around 1 million generations ago (Fig. 1*E*). We could detect no signal of bottleneck, in particular no bottlenecks associated with climatic fluctuations, consistent with *B. fruticulosa*’s endemic status in a major Mediterranean refugium (34). Combined with moderate and steady effective population sizes, this indicates neither drift nor bottleneck underlies the contrasting phenotypic, transcriptomic, and genome scan patterns we detail below.

### Contrasting Functional Diversity in Salinity Responses at the Local Level.

Given our recognition of two larger genetic clusters, consistent with local origin (here termed the “North” and “Central” metapopulations) we performed a salinity challenge experiment to test the transcriptome response of each population. We chose the most sharply contrasting populations from each major genetic cluster (North and Central) from the hydroponic salinity challenge experiment above and then subjected them to salinity (0 mM or 150 mM NaCl) for 10 d. Leaves and roots of six plants per population [48 samples total: two tissues of 24 plants from two salt-tolerant (ST) and two salt-sensitive (SS) populations, 12 plants under control and 12 plants under salt stress conditions] were sequenced for transcriptome analysis.

**Fig. 3.**
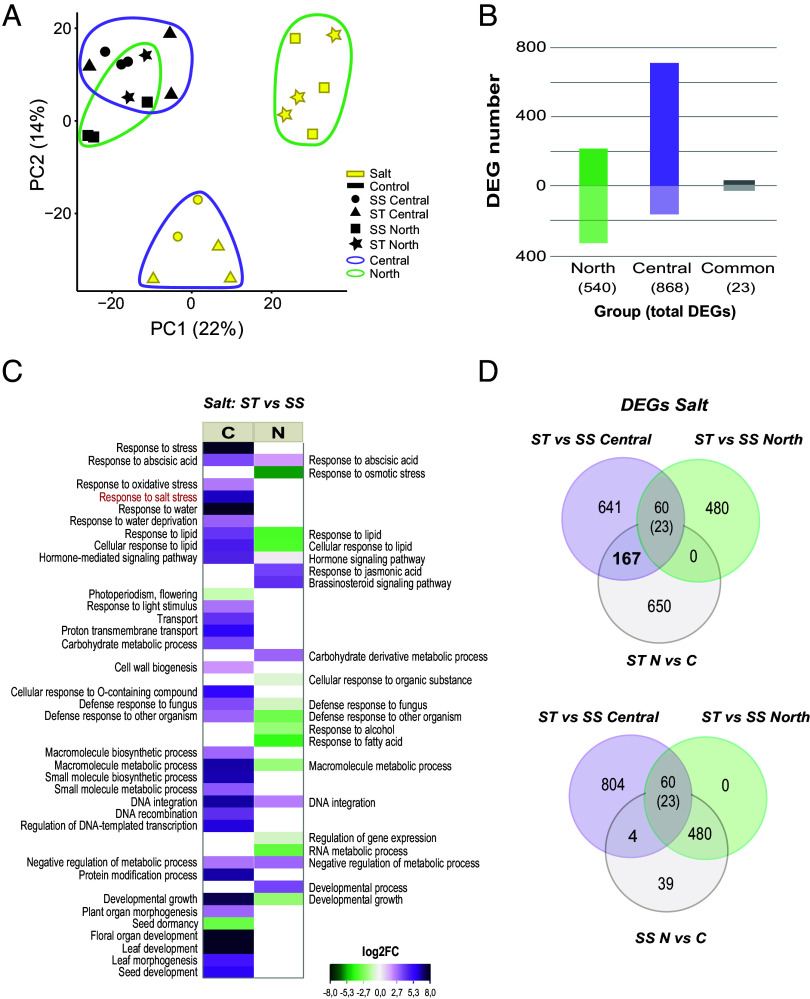
Differential transcriptome responses to salinity challenge between *B. fruticulosa* North and Central metapopulations. (*A*) PCA of leaf transcriptome profiles of 11 ST (triangles/stars) and 11 SS (circle/square) *B. fruticulosa* individuals from North (green outline) and Central populations (purple outline) treated with 0 mM NaCl (control, black) or 150 mM NaCl (salt, yellow) for 10 d. Root data are given in *SI Appendix*, Fig. S2. (*B*) Number of differentially expressed genes (DEGs) in pairwise comparisons ST vs. SS under salt stress of North populations (green bars), Central populations (purple bars), and shared in both origins (gray bars). (*C*) Significantly enriched gene ontology (GO) terms (biological process category, Fisher’s exact test, *P*-value < 0.05) associated with DEGs from ST vs. SS North comparison (*Right*), and from ST vs. SS Central comparison (*Left*). Scale color indicates the L2FC mean of the genes included in each GO term. (*D*) Venn diagrams of DEGs shared when analyzing ST vs. SS individuals from the North or the Central populations and when analyzing North vs. Central individuals from ST or SS populations, always in plants under salt stress. The overlap is significantly greater than expected by chance (Permutation test, *P* < 0.05).

PCA of the overall transcriptome response revealed a marked differential effect of genetic cluster on salinity response profiles, specifically in leaves (*SI Appendix*, Fig. S2). While under control conditions, all samples cluster together regardless of population origin or salt sensitivity (black shapes in Fig. 3*A*), upon salt treatment (yellow shapes in Fig. 3*A*), plants from the North (circled in green) and plants from the Central (circled in purple) show divergent transcriptomic responses along both PC1 and PC2. This points to a specifically salinity-related divergence in genomic responses between these two salt-resistant metapopulations at the transcriptomic level. This also provided a hint at divergent salinity tolerance mechanisms between the North and Central metapopulations, despite close proximity in nature and very low genetic differentiation between the North and Central coastal populations (pairwise fixation index (Fst) = 0.13).

Considering this contrasting salinity response in each group revealed by PCA, we analyzed transcriptome responses separately for North or Central populations, hypothesizing that ST plants might be using different strategies to cope with salinity stress. Doing so, we found that the quantity and identity of differential expressed genes (DEGs) differed greatly between the North and Central metapopulations. The analysis of ST vs. SS *B. fruticulosa* plants from the North populations (green in Figs. 2 *D*–*F* and 3*B*) gave 540 DEGs (L2FC > |2|, *P*-adj < 0.05) activated in leaves only under salt stress conditions, with 213 up- and 327 down-regulated. In the same analysis performed with the Central populations (purple in Figs. 2 *D*–*F* and 3*B*) we obtained a total of 868 DEGs, 708 up- and 160 down-regulated under salinity (Dataset S6). Many fewer DEGs shared the same expression pattern in both analyses (only 23 DEGs; corresponding to 2.6% of DEGs in Central and 4.3% of DEGs in Northern), which constitutes a very small minority of the DEGs in each cluster (Fig. 3*B*). This confirms the observation of contrasting global transcriptional salinity stress responses as a function of genetic clusters by PCA (Fig. 2*C*).

Both the greater quantity of up-regulated genes detected in the Central cluster, together with the gene ontology (GO) terms enriched (Fig. 3*C* and *SI Appendix*, Figs. S3 and S4*A*), indicate that salinity stress activates several biological responses to cope with abiotic stresses specifically in the Central populations: transport control, hormone regulation, decrease of oxidative stress, and responses to water deprivation. The transcriptomic analysis (Dataset S6) provides valuable information guiding future characterization of the genes potentially involved in salt tolerance based on internal Na^+^ detoxification in the *B. fruticulosa* populations from the Central cluster. Here, we point out only a few examples of highly up-regulated genes of interest, based on homology to *A. thaliana* and other Brassica relatives where these have been studied. Involved in the alleviation of oxidative stress, among others, we find glutathione transferases (*GSTU3*, 6, 11, 13, and 25) and alternative oxidases *AOX1A* and *AOX1B* (22- and 3-fold, respectively); in transport the vesicle transporter SEC1A (20-fold), the fluoride exporter *FEX* (sixfold), the Mg transporter *AT2G04305.1* (sevenfold), and the vacuolar transporter *ABCG19* (ninefold) are notable. In stomatal regulation and drought and salt responses *DUF810* (20-fold) and *YL1/BPG2* (11-fold), both involved in salt stress through *ABI4* regulation, stand out.

In contrast, most of these stress response terms are not enriched, or are down-regulated, in the North populations under salinity challenge (Fig. 3*C* and *SI Appendix*, Figs. S3 and S4*A*). This indicates that, relative to ST Central plants, ST Northern plants are not sensing stress caused by Na^+^ toxicity. Indeed, when we analyze the DEGs detected in the North vs. Central comparisons with the DEGs from the SS*vs*ST comparison of the North populations, the majority of DEGs belong to the SS (inland) *B. fruticulosa* populations (Fig. 3*D*). This suggests that in the Northern populations, the strongest response to salt stress occurs in the stressed/inland plants and therefore the tolerance mechanism of the tolerant/coastal plants is partially masked. However, secondary metabolism and hormonal signaling pathways are clearly activated through upregulation of *ERF* transcription factors (TF) (*ERF/AP2*, sixfold) and other genes involved in ABA (*EDL3*, 10-fold) and Brassinosteroid (*BR6OX2*, fourfold) signaling. Other examples of strongly up-regulated genes in the tolerant Northern cluster are, among others, sterol isomerase (*AT1G05440*, ninefold); boron efflux transporter *BOR4* (sevenfold); *SNF1*, involved in osmotic and ionic stress response (sevenfold); or *OXR1*, an oxidative stress tolerance gene (sevenfold) (Dataset S6).

**Fig. 4.**
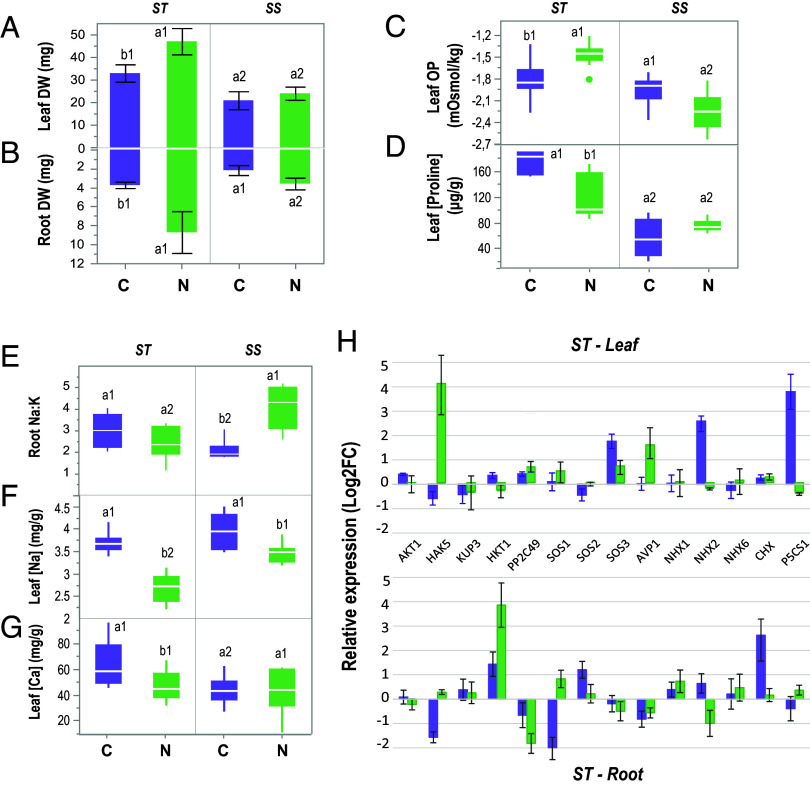
Disjunct adaptive phenotypic and genotypic responses of North and Central *B. fruticulosa* populations. (*A*) Shoot and (*B*) root biomass (DW, g); (*C*) leaf osmotic potential (mOsmol/Kg), (*D*) leaf [proline] (µg/g FW), (*E*) root Na:K ratio, (*F*) leaf [Na^+^] (mg/g) and (*G*) leaf [Ca^2+^] (mg/g DW) of SS and ST *B. fruticulosa* plants from North (green) and Central (purple) populations cultivated hydroponically and treated with 0 mM NaCl (control) or 150 mM NaCl (salt) for 10 d. In panels, (*A*–*G*) letters indicate significant differences between geographic origin (C;N) and numbers indicate significant differences between salinity tolerance phenotype (ST;SS) (ANOVA, *P* < 0.05). (*H*) Root and leaf relative expression (Log2FC salt vs. control) of 14 orthologs involved in Na^+^ and K^+^ transport or salinity responses in ST *B. fruticulosa* plants.

To better resolve potential mechanisms used by ST Northern and Central plants, salt stress-diagnostic physiological parameters were evaluated in the same individuals used for the transcriptome analysis. Overall, ST plants maintained higher osmotic potential and exhibited greater growth than SS plants, independently of cluster origin (Fig. 4 *A*–*C*). However, ST Northern plants were much more efficient at restricting Na^+^ translocation and favoring K^+^ uptake than ST Central plants (Fig. 4 *E* and *F*). Indeed, when we tested expression levels of well-studied orthologs involved in Na^+^ and K^+^ transport, we observed that *HKT1* was significantly up-regulated especially in the roots of ST plants from North populations (Fig. 4*H*). It has been seen in several species that high expression levels of HKT transporters in the root lead to lower shoot Na^+^ concentrations due to the role of *HKT* in retrieval of Na^+^ from the transpiration stream (35). Also, a repressor of *HKT1*, *PP2C49*, was down-regulated (Fig. 4*H*), probably enhancing shoot Na^+^ extrusion, as shown in *A. thaliana pp2C49* mutants (36). In addition, high salinity usually causes the downregulation of *HAK/KUP/KT* family members such as *HAK5* in roots (37). However, in ST Northern *B. fruticulosa* plants the expression of *HAK5* in roots was not altered and the gene was up-regulated in the leaf tissue (Fig. 4*H*), suggesting that K^+^ uptake and translocation may be promoted preventing Na^+^ accumulation.

Contrastingly, ST Central plants accumulated higher Na^+^ levels in the leaves, but the osmotic imbalance was compensated with a higher production of compatible solutes such as proline (Fig. 4*D*). Influx of Na^+^ into the root system is counteracted by efflux of Na^+^ to the rhizosphere, an active process that occurs in antiport with H^+^. This process involves the *Salt Overly Sensitive* (*SOS*) pathway in many plant species, in which the calcium-binding protein *SOS3* recruits the Ser/Thr protein kinase *SOS2* to the plasma membrane where it activates the Na^+^/H^+^ antiporter *SOS1* by phosphorylation (38). *SOS1* was down-regulated in ST Central roots (Fig. 4*H*), showing the inverse relationship between *SOS1* expression in the root and total plant Na^+^ accumulation also found in other brassicas (39, 40). Thus, the Na^+^ efflux in ST Central plants is low but they must have the ability to tolerate Na^+^ inside their tissues. Vacuolar storage of Na^+^ is mediated by vacuolar Na^+^/H^+^ antiporters (*NHX* clades) and the electrochemical potential is provided by vacuolar H^+^-pyrophosphatases (e.g., *AVP1*) and vacuolar H^+^-ATPase (41). Indeed, *NHX2* was significantly up-regulated in the leaves of ST Central plants (Fig. 4*H*) and could be enhancing Na^+^ vacuolar storage. Nonetheless, we also detected an overexpression of a cation/H^+^ exchanger (*CHX*), a putative K^+^, Na^+^/H^+^ antiporter, in the roots of ST Central plants (Fig. 4*H*). The *CHX* genes of *A. thaliana*, soybean, rice, and other plants have been identified as being involved in tolerance to salt stress thanks to their role in K^+^ and Na^+^ acquisition and homeostasis (42). Recently, Guo et al. (43) showed that overexpression of a halophyte *CHX* gene (*KvCHX*) in *A. thaliana* seedlings enhanced their salinity tolerance by increasing K^+^/Na^+^ and proline levels.

ST Central plants also harbored elevated Ca^2+^ levels in leaves (Fig. 4*G*), suggesting that Ca^2+^ waves and root-to-shoot signaling are more active in these plants. Salt stress and changes in osmotic pressure are associated with the elevation of Ca^2+^ in the cytosol and the activation of ROS signaling. These signals induce adaptive processes to alleviate Na^+^ toxicity, including the maintenance of ion balance, the induction of phytohormone signaling, and increased synthesis of osmolytes and antioxidant enzymes (44, 45). Indeed, the orthologue of *P5CS1* (a synthase that catalyzes the rate-limiting enzyme in the biosynthesis of proline), was highly expressed in ST Central plants (Fig. 4*H*). P5CS1 appears to be involved in salt stress responses related to proline accumulation, including protection from reactive oxidative species (46), and may be crucial for *B. fruticulosa* salinity tolerance.

### Selection Signatures Associated with Contrasting Saline Coastal Adaptation.

**Fig. 5.**
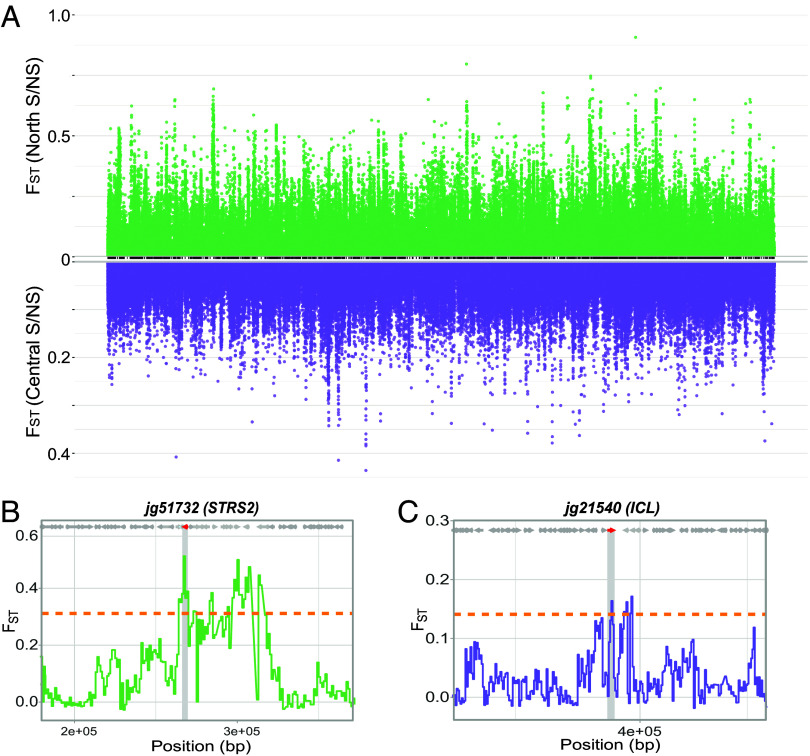
Contrasting selection signatures in two adjacent saline-adapted *B. fruticulosa* metapopulations. (*A*) Contrasting patterns of ecotype-specific differentiation across the *B. fruticulosa* genome. Top Manhattan plot (green) shows FST values between coastal and inland populations in the North genetic cluster; bottom Manhattan plot (violet) shows FST values between coastal and inland populations in the Central genetic cluster on an inverse *y*-axis in order to closely illustrate completely selective signals across the entire genome (*x*-axis). Estimates of FST for coastal selection candidates from the North (*B*) and Central (*C*) clusters. Solid lines give localized elevated FST, which is accompanied by low Tajima’s D driven sharply negative in the focal, coastal population, a classic signal of selection. All genes are FST 1% outliers related to salinity, jg51732 (AT5G08620) encodes a *STRESS RESPONSE SUPPRESSOR 2* (*STRS*2) that binds to RNA and is involved in drought, salt, and cold stress responses; jg21540 (AT3G21720) encodes a glyoxylate cycle enzyme IsoCitrate Lyase (*ICL*) involved in salt tolerance.

Having established that local adaptation to coastal environments is associated with two contrasting adaptive salinity tolerance strategies, we sought to further determine which alleles might underlie these phenotypes. Adaptation to coastal environments is complex and multidimensional, so we expect a diversity of selective pressures; further, given our differential salinity adaptation phenotypes between the North and Central clusters, we expect particular regions to vary. We therefore applied a diversity of parametric and nonparametric tests for selection. We first leveraged our population resequencing to search for footprints of selection between coastal and inland populations. Given the extremely low within-group genetic differentiation (mean saline/nonsaline Fst = 0.016 to 0.019) and the lack of bottleneck (Fig. 2*E* and Dataset S5), this approach should avoid artifacts caused by bottlenecks or excess population differentiation. For each cluster (North and Central), we measured Hudson’s Fst in 1 kb windows between saline and nonsaline populations and from this extracted the 1% extreme outliers from the empirical distribution; these were reserved as our “Coastal Selection Candidates” (Fig. 5, *SI Appendix*, Fig S4*B*, and Dataset S8).

We then focused on particular soil elements that significantly vary between coastal and inland sites (soil Na and Mg, Dataset S2), using these discriminant soil ion values as phenotypes in a targeted EAA. Here, we inferred candidates directly associated with the chemical characteristics of each sequenced plant’s immediately proximal soil by performing EAAs using Latent Factor Mixed Model (LFMM2) (47). This analysis quantitatively determines the association between each soil element concentration and SNPs across the genome in all populations at the level of individual plants (North = 30; Central = 44). We identified 433 (North) and 56 (Central) genes harboring ≥1 SNP significantly associated with at least one distinctive soil parameter identified (“EAA Outliers,” Dataset S9).

Finally, we performed a genome scan for regions of the genome that are outliers vis-à-vis background demographic structure with *PCAdapt* (48). Such an approach provides an orthogonal test relative to the above, as it uses no priori information regarding group structure (as Fst scans do) or proposed selective agent/correlate (as EAAs do), instead identifying outlier regions based on a genome-wide Mahalanobis test statistic (“PCAdapt Outliers,” Dataset S10).

Each of these approaches (transcriptome response, Coastal Selection Candidates, EAA outliers, and PCAdapt outliers) yielded relevant candidates (Datasets S6–S10). But, as these approaches focus on orthogonal signals, there was limited concordance (*SI Appendix*, Fig. S5). However, we do observe overlap at the level of GO enrichment within each genetic cluster. This overlap was significantly greater than expected by chance, as determined by permutation tests (*P* < 0.05, Dataset S11). Common relevant GO terms indicated by multiple approaches include “response to osmotic stress” in the North cluster, and “response to water deprivation” and “response to salt stress” in the Central cluster, both relevant to salinity adaptation (*SI Appendix*, Fig. S5 *C* and *D* and Dataset S11). Candidate genes contributing to these categories in our top candidate lists include the *Plasma membrane Intrinsic Protein 1* (*PIP1*) and *Stress Response Suppressor 2* (*STRS2*) for the North cluster and *Tonoplast Intrinsic Protein 2* (*TIP2*), *Salt Tolerance 32* (*SAT32*), *Sugar Insensitive 8* (*SIS8*), *UDP-Glycosyltransferase 79* (*UGT79B2*), and *Isocitrate Lyase* (*ICL*) for the Central cluster. Membrane and tonoplast aquaporins such as *PIP1* and *TIP2* play crucial roles in mitigating salinity stress by maintaining water and ionic homeostasis and better cell membrane integrity (49). Mutations in the *DEAD-box RNA helicases STRS1* and *STRS2* caused increased tolerance to salt and osmotic stress in *A. thaliana* by attenuating the expression of stress-responsive transcriptional activators (50). Curiously, *SAT32*, associated with salt tolerance and ABA signaling in *A. thaliana*, has six copies and high structural variation in the halophytic *Eutrema salsugineum* (51). *A. thaliana SIS8* is a MAPK that interacts with UDP-glucosyltransferase in the nucleus and negatively regulates salt tolerance (52) while overexpression of *UGT79B2/B3* enhanced plant tolerance to salt stresses modulating anthocyanin accumulation (53). In rice, *ICL* is regulated by a calcium sensor calmodulin-encoding gene. The overexpression of both confers salinity tolerance in *A. thaliana* and rice by shifting energy metabolism from the tricarboxylic acid (TCA) cycle to the glyoxylate cycle during salt stress (54).

### Evapotranspiration as a Potential Selective Agent in a Hyperlocal Scale.

It was straightforward in *B. fruticulosa* to demonstrate local adaptation to coastal conditions as well as specific tolerance to elevated salinity. But we note also that adaptation to coastal environments is complex and multidimensional, with challenges to colonists not only from elevated salinity, but also various nutrient deficiencies and dehydration due to increased substrate porosity, wind exposure, sand-loaded sea spray, and high light intensity among others (55). Accordingly, signatures of selection may be expected to originate from stressors both common across coastal sites, but also distinctive to particular regions and even at the very local, microsite-scale [e.g., the scale of tens of meters; (56)]. Thus, given the markedly contrasting genomic, physiological, and adaptive responses to the primary selective agent, salinity, we finally sought whether there may be subtle local differences in environmental challenge that may drive divergent responses to the ostensible primary agent of coastal salinity that were less obvious than the strong coastal salinity gradient across this region. Classic literature indicating a hyperlocal scale of plant adaptation further suggests fine-scale responses to stressors in adjacent plant populations, e.g., on coastlines or mines (57). These works found that especially in sedentary plants, selection can cause extremely localized patterns of microgeographical adaptive variation despite a degree of migration; however, these processes depend on various ecological and genetic factors that are very challenging to evaluate, and unique for each species and habitat.

**Fig. 6.**
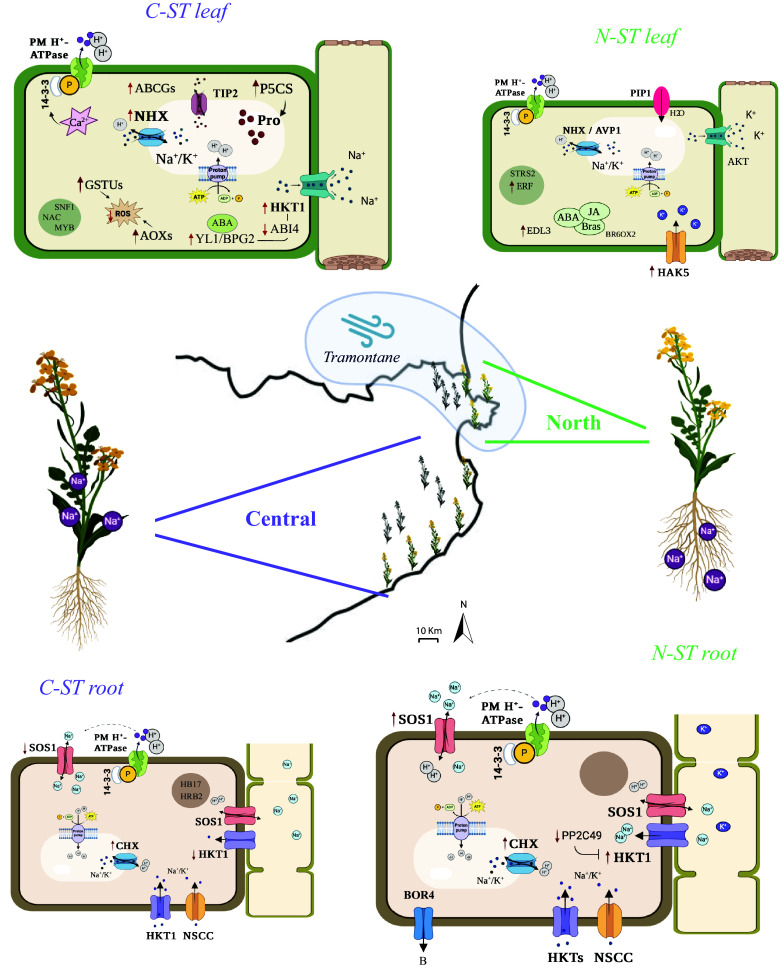
Overview of the contrasting salinity tolerance strategies of the North and Central *B. fruticulosa* coastal metapopulations. Hypothetical model of genes, ion transport, and signaling pathways involved in salinity tolerance mechanisms. Gene symbols are shown in bold letters. Ion fluxes are indicated with black arrows. Gene activation/repression and molecule increase/decrease are indicated with red arrows. Star-framed symbols denote signaling pathways and hormone molecules are circled in green. “ST” = Salt tolerant.

In our study region soil elemental composition does not vary between North and Central coastal locations (Dataset S2); therefore, we looked more closely at climatic data, despite the highly local scale. While precipitation and daily temperatures did not differ between regions in the 14 y evaluated, evapotranspiration did (*SI Appendix*, Fig. S6 and Dataset S12). Evapotranspiration (ET_0_) is the sum of plant transpiration and soil evaporation and is affected by many environmental factors such as net radiation, air temperature, relative humidity, and especially wind speed (58). Indeed, relative to the Central region, tramontane wind is prevalent in the far North of Catalonia, and this explains why annual mean ET_0_ was significantly higher there, despite no substantial difference in precipitation or daily temperatures (*SI Appendix*, Fig. S6 and Dataset S12). An increased evaporative demand tends to increase transcriptional volume flow, leading to greater salt damage on plants (59). Under these circumstances, we speculate that the observed Na^+^ exclusion strategy of the North cluster may be more effective. We theorize therefore that harsh coastal conditions aggravated by high ET_0_ are the primary agents of selection affecting *B. fruticulosa* Northern region plants, resulting in selection on genes that confer advantage under drought and salinity stress. Contrastingly, in the Central region, water shortage is less severe, and plants can cope with salinity with tissue tolerance and osmoprotection strategies (Fig. 6). In future, the genes and signaling pathways discussed in this study should be validated with metabolomic and mutant assays that evidence the implication of particular markers in these two contrasting salinity tolerance strategies.

## Conclusions

Here, we set out to develop a model of within-species salinity tolerance in an outcrossing Brassicaceae, first by an exhaustive survey of 13 species in an ecologically well-understood region coastline in Northeast Spain. This search resulted in the identification of two distinct evolved strategies of salinity tolerance in adjacent metapopulations of the same species, *B. fruticulosa* (Fig. 1). These metapopulations genetically cluster in three groups regardless of their coastal or inland origin and deploy contrasting mechanisms to mitigate salinity stress on the order of a dozen kilometers (Fig. 2). Relative to Central ST plants (C-ST), Northern ST plants (N-ST) excluded Na^+^ to a higher degree, restricting its translocation to the aerial tissues and favoring K^+^ uptake (Figs. 4 and 6). After 10 d under high salinity, root growth and synthesis of secondary metabolites were stimulated, key ion transporters such as *SOS1* and *HKTs* were respectively down- and up-regulated in roots, and salt-responsive genes were not activated in leaves, suggesting that N-ST plants were not engaged in a salinity stress response (Figs. 3, 4, and 6). In contrast, C-ST did not restrict Na^+^ translocation, and accordingly instead activated the osmoprotectant machinery to compartmentalize Na^+^ in the vacuoles, thus reducing oxidative damage (Figs. 3, 4, and 6). These divergent salinity responses were evidenced also in a variety of genome scans for the genomic basis of these contrasting responses (Fig. 5). We identify candidates for a Na^+^ exclusion/extrusion system and for an effective Na^+^ compartmentalization mechanism, two strategies highly relevant for cultivars that need to adapt to the new environmental inclemencies. This work therefore establishes *B. fruticulosa*, a wild relative diploid compatible with crops such as *B. rapa*, *Sinapis alba,* or *Raphanus sativus*, as a promising source of wild desirable alleles, as well as these highly diverse natural populations as powerful models for the study of adaptations to saline soils.

Our observed contrast in evolved ecophysiological salinity tolerance strategies between the North and Central metapopulations is an interesting problem. These populations are situated on a coastal strip with a marked uniform salinity gradient, acting as obvious selective agents across the study region. However, this clearly divergent adaptive response of two adjacent populations required us to question whether other latent stressors may modify primary adaptive strategies. While soil salinity appears to be the principal agent driving coastal adaptation of *B. fruticulosa* populations, we speculate that other microclimate factors like variation in evapotranspiration rates may drive contrasting salinity tolerance strategies (Fig. 6). Future studies should work toward fully decoupling the complex selective forces responsible for these contrasting strategies.

The divergent salinity adaptation strategies we detail here, each with different genetic and mechanistic underpinnings, are perhaps surprising. We suggest that this may be less surprising than the result of it is a modern prediction that has not sufficiently been empirically tested because of inadequate sampling, technology, or the absence of this experimental/conceptual lens (but see ref. 8). The examples we detail here emphasize the idea that salinity tolerance amounts to an emergent manifestation of complex underlying networks that propagate up through all the omics. They show how contrasting strategies get to similar outcomes, each perhaps subtilty tuned to fine-scale environmental differences. This then leads to the expectation that the convergent phenotype at one level (“salinity tolerance”) will have many possible origins at the level of mechanism.

## Materials and Methods

### Plant and Soil Materials.

Seeds of wild Brassicaceae species were collected in Spring 2017 across Catalonia (map: https://www.google.com/maps/d/u/0/edit?mid=1PGHyv3p7AwtI57N6C_eC6ecbnj0&usp=sharing). Each population was classified depending on coastal (<3 km from the sea) or inland origin. The six brassica species with two or more inland and coastal populations were selected for testing salinity tolerance (*B. fruticulosa*, *L. graminifolium*, *L. maritima*, *D. tenuifolia, D. erucoides*, and *D. muralis*). Seeds were stored in cold and dry conditions until use.

Leaves of *B. fruticulosa* plants from 19 populations were collected and brought to the lab. Plants from these populations were confirmed diploid. Leaf samples from six individuals per population were dried for 48 h at 60 °C and stored in a drier for the following ionomic analysis. Leaf samples from five individuals per population were frozen and kept at −20 °C for subsequent DNA extraction and sequencing. Soil samples from near three *B. fruticulosa* plants separated at least 2 m were collected for each of the 19 populations, air-dried for 2 d, 2 mm sieved, and stored in cold/dry conditions for further soil ionomic analysis.

### Soil Cultivation of Brassica Species.

Seeds of six Brassica species (*B. fruticulosa*, *L. graminifolium*, *L. maritima*, *D. tenuifolia*, *D. erucoides*, and *D. muralis*) were sowed in 8 × 8 × 8.4 cm square pots filled with common potting mix soil (20 pots per species), distributed randomly in trays and placed in a controlled environment room (12 h light, 22 °C ± 2 °C). Five days after germination, one plant per pot was left. Plants were irrigated with ¼ Hoagland solution every 2/3 d. Seven days postgermination, 3/4 of the plants (15 pots per species) were gradually irrigated with 25 mM of NaCl, 1/3 of them reaching 100 mM of NaCl and collected at 21 d old, 1/3 reaching 200 mM of NaCl and collected at 32 d old, and 1/3 reaching 300 mM of NaCl until maturation. The aerial tissue of each plant was dried for 48 h at 60 °C and weighed to obtain the aerial biomass.

### Reciprocal Transplant Experiment.

*B. fruticulosa* seeds from three coastal (BLA, ROS, TOS) and three inland (PAU, TOR, SOL) populations were sowed in uncultivated fields of the Marimurtra Jardí Botànic de Blanes (JBB: 41.677320, 2.801198), a representative coastal environment, and of Les Planes d’Hostoles (LP: 42.064403, 2.544788), a representative inland environment. The same common garden design was reproduced at both sites, with 10 individuals per population planted into 20 × 20-cm squares. One leaf of three 30-d-old plants per population was harvested and dried for ionomic analysis. Fitness was evaluated counting the number of siliques at maturity.

### Germination Tests and Hydroponic Cultivation.

*B. fruticulosa* seeds from nine coastal (BEG, BLA, ESC, GAR, LAN, POR, ROS, SFG, TOS) and nine inland populations (BRU, FUM, LOF, PAL, PAU, PiM, SCF, SOL, TOR) were first soaked in 30% bleach solution (30% commercial bleach (2% NaOCl), 70% deionized (dd) water, and two drops of Tween® 20) for 15 min, then washed with ddH_2_O 5 times, and finally suspended in 10^−5^ potassium nitrate solution. Seeds were stratified for 2 d at 4 °C to synchronize germination. Thirty seeds from each population were placed in square plates with ½ Murashige-Skoog (MS) medium (pH 6, Control) or ½ MS medium with 75 mM of NaCl (pH 6, Salt) to test the germination rate. Plates were placed in a growth chamber with 150 mmol/m^2^s of light intensity, 12 h light/12 h dark photoperiod, and 25 °C d/night temperature.

One-week-old seedlings from the control plates were transferred to a hydroponic system filled with 0.5-strength Hoagland solution (pH 6, Control) in the same growth chamber. The hydroponic solution was changed every 2 to 3 d to maintain a constant concentration of nutrients in the solution and in half of the plants NaCl was increased gradually (50 mM NaCl every 2/3 d) until a final concentration of 150 mM of NaCl (pH 6) when plants were 21 d old. Ten days later, we harvested the plants and measured plant growth parameters (root length, biomass). Plants from four coastal (BLA, ESC, ROS, TOS) and four inland *B. fruticulosa* populations (PAU, SCF, SOL, TOR) were selected to measure osmotic potential, proline, and root and leaf ionome. Samples for RNA extraction were immersed in liquid nitrogen, homogenized to a fine powder, and stored at –80 °C.

### Soil and Plant Tissue Ionomic Analysis.

To characterize the elemental composition of soil, analyses were performed on the 2-mm fraction samples following the extraction method described in Busoms et al. (9). Plant tissue was dried for 2 d at 60 °C. Approximately 0.1 g was weighed and used to perform open-air digestion in Pyrex tubes using 0.7 mL concentrated HNO_3_ at 110 °C for 5 h in a hot-block digestion system (SCI54-54-Well Hot Block, Environmental Express, Charleston, SC, US). Concentrations of the following elements (Li, B, Ca, K, Mg, Na, P, S, Mo, Cu, Fe, Mn, Co, Ni, Zn, Cr, As, Rb, Sr, Cd, and Pb) were determined by Inductively coupled plasma mass spectrometry (ICP-MS) (Perkin Elmer Ink., ELAN 6000, MA, US) or ICP-OES (Thermo Jarrell-Ash, model 61E Polyscan, UK). Mean‐standardized values (1 < value > 1) of elemental contents were used to represent the radar plots.

### Proline Quantification.

Proline concentration was determined colorimetrically from frozen tissue using a method adapted from Bates et al. (60). Briefly, fresh plant material (50 mg) was homogenized with 3% 5-sulfosalicylic acid and centrifuged at 10,000 rpm, 4 °C for 10 min. Supernatant was collected and stored at −20 °C. For the reaction mixture preparation, ninhydrin was dissolved in hot glacial acetic acid (1:24 w/v) and then carefully mixed with 6 M orthophosphoric acid (3:2 v/v). In crystal tubes, glacial acetic acid, ninhydrin mix, and plant extract was mixed (1:1:1 v/v/v) and subjected to a hot water bath at 98 °C for 60 min. Then, samples were cooled down in ice for 20 min and toluene was added and vortexed for 20 s. Finally, the organic phase was extracted, and the absorbance was measured at 520 nm. Proline concentration was calculated using a calibration curve.

### Osmotic Potential.

Frozen tissue (0.2 g) was placed into a 0.5 mL Eppendorf and boiled in a water bath for 30 min. The resulting liquid was extracted, centrifuged 10 min at 15,000 rpm, and the supernatant was measured in a freezing-point depression osmometer (Osmomat 3000, Ganotech).

### Oxford Nanopore Sequencing, Genome Assembly, and Annotation.

High molecular weight (HMW) DNA was isolated from one plant from Tossa de Mar (TOS), Catalonia, Spain. This plant was confirmed diploid (*SI Appendix*, Fig. S1). HMW DNA was extracted from 2.5 g of leaves that had been etiolated for 24 h, according to the nuclear DNA isolation protocol published by Bernatzky and Tanksley (61), with some modifications. In brief, leaves were snap frozen in liquid nitrogen, ground to a fine powder, homogenized in extraction buffer (0.35 M Sorbitol, 100 mM Tris, 5 mM Ethylenediaminetetraacetic acid (EDTA) (pH 7.5) with 1% β-mercaptoethanol added freshly before use) by vortexing for 30 s. The resulting slurry was filtered through Miracloth and centrifuged for 15 min at 720 g. The pellet was resuspended in extraction buffer containing 0.4% Triton X-100 and centrifuged for 15 min at 720 g. This step was repeated a second time. The pellet, containing nuclei, was resuspended in extraction buffer containing 100 μg/mL of RNase, and nuclei were lysed by mixing the suspension with CTAB Lysis Buffer (ITW Reagents; A4150) and adding 1/6 volume of 5% N-laurylsarcosine. The lysis mix was incubated at 65 °C for 20 min, before the lysate was mixed with 2.2 volumes of chloroform/isoamylalcohol (24/1) and centrifuged at 5,000 g for 15 min. The aqueous phase was mixed with an equal amount of cold isopropanol to precipitate the DNA. The DNA was hooked out of the solution with a glass rod, washed by dipping in 80% ethanol, and then resuspended in 100 μL of TE buffer (10 mM Tris-HCl, 1 mM EDTA; pH 8). Genomic DNA was left to resuspend overnight, then quantified using the Qubit Fluorometer and the Qubit DNA dsDNA BR Assay (ThermoFisher; Q32853). DNA (1 μg) was used as the input for an ONT ligation sequencing library preparation (ONT; SQK-LSK109). The library was quantified using the Qubit Fluorometer and the Qubit dsDNA High Sensitivity (HS) Assay Kit (ThermoFisher; Q32854). 650 ng of library was sequenced over one PromethION flow cell (ONT; FLO-PRO002) and run on a PromethION Beta sequencer.

Fast5 sequences were basecalled using Guppy [version 6 high accuracy basecalling model dna_r9.4.1 _ 450bps_hac.cfg; (62)] and the resulting fastq files were quality filtered by the base caller. For assembly, we used 1,806,322 ONT reads with a read length N50 of 31 kb (21 total GB ONT data). Base called fastq files were assembled using Flye [version 2.9; (63)] at a depth of 38×, assuming a 557 megabase genome [Kmer-based genome size estimates were performed with FindGSE (64)] into a pseudohaploid primary assembly. Contigs were then polished to improve the single-base accuracy in a single round of polishing with Medaka [version1.5.0; (65)] using the ONT long reads, followed by a second round of polishing incorporating paired-end Illumina HiSeq data with Pilon (version 1.24) (66). We purged uncollapsed duplicate haplotypes using PurgeDups (67). We removed the contaminating sequences by using the BlobTools pipeline (68) using coverage, Diamond-blast, and GC content information, with results shown in Dataset S5. We then used the BRAKER pipeline to conduct gene annotation on the genome assembly (29). Evidence types included RNAseq data (generated as below) and protein data from related species.

### Contamination Removal and Assembly Assessment.

Removal of contamination from the assembly was performed in Blobtools (68), based on phylogenetic assignment, copy number, and GC content. The Uniprot database was downloaded (8 May 2022) and the assembly was blasted against this using (Diamond version 2.0.15) (69). Gene space completeness was assessed using BUSCO version 5.2.2 (70) and the odb10 database for Brassicales, employing default parameters.

### Short-Read Library Preparation and Sequencing.

Genomic DNA was isolated from *B. fruticulosa* young leaves using the Wizard® Genomic DNA Isolation Kit. Quantification of DNA was performed using Quant iT dsDNA HS Assay (Thermo Fisher; Q-33120) with a Fluoroskan Ascent Fluorometer (Thermo Fisher; 5200111). Illumina DNA sequencing libraries were constructed using the Illumina DNA Prep library preparation kit (Illumina; 20018705) with IDT® for Illumina Unique Dual indexes, sets B and D Library preparation was performed using a Mosquito HV (SPT Labtech) liquid handling robot with 1/10th volumes at all steps. A total of 9 to 48 ng of DNA was used as library input and five cycles of PCR were used for the library amplification step. Final libraries were normalized and pooled based on quantification performed by Quant iT dsDNA HS Assay (Thermo Fisher; Q-33120) on a Fluoroskan Ascent Fluorometer (Thermo Fisher; 5200111). The resulting pools were size selected using 0.65× AMpure XP Beads (Beckman Coulter; A63882) to remove library fragments <300 bp. Size selected pools were checked for size distribution on HS D1000 ScreenTape (Agilent; 5067 to 5584) using an Agilent TapeStation 4200 and quantified using Qubit™ 1× dsDNA HS Assay on a Qubit 4 Fluorometer. Final library pool quantification was performed using the KAPA Library Quantification Kit for Illumina (Roche; KK4824). The libraries were sequenced with Illumina NovaSeq 6000 at Novogene Europe, Cambridge, UK.

### Short-Read Processing and Variant Calling.

Low-quality reads and sequencing adapters were removed using Trimmomatic [PE -phred33 LEADING:10 TRAILING:10 SLIDINGWINDOW:4:15 MINLEN:50; (71)] and the surviving reads aligned to the *B. fruticulosa* reference genome using bwa-mem2 (72). We removed duplicated reads using Picard tools (https://broadinstitute.github.io/picard/) and identified SNPs using GATK4 (73). Filtering of the variant calls was based on the GATK’s best practice guidelines, and we included filters for mapping quality (MQ^3^ 40 and MQRankSum^3^ –12.5), variant confidence (QD^3^ 2), strand bias (FS < 60), read position bias (ReadPosRankSum^3^ –8), and genotype quality (GQ^3^ 10). We further removed SNPs with sequencing depth 1.6× the mean depth to avoid issues caused by paralogous mapping.

### Analysis of Genetic Structure and Variation.

All genetic structure and demographic analyses were run using fourfold degenerate sites identified with DEGENOTATE (https://github.com/harvardinformatics/degenotate). Performance was verified by running SNPeff to confirm the presence of only synonymous variants. For fastSTRUCTURE analysis we LD thinned in 5 kb windows allowing no more than 20% missing data and minimum allele frequency of 2.5% to yield a dataset of 30,272 fourfold degenerate sites. fastSTRUCTURE was then run at K1 to K10 five times to clarify the extent of gene flow between populations. For PCA and SplitsTree analysis of Nei’s genetic distances, we thinned the dataset with a custom C script prune_ld (74) that thinned to *r*^2^ ≤ 0.1 in 100 SNP windows also allowing no more than 20% missing data and minimum allele frequency of 2.5% which yielded a dataset of 19,689 fourfold degenerate sites. We then conducted a PCA on the fourfold dataset ([MAF] > 0.05). Following Patterson et al. (75), we estimated a covariance matrix representing the genetic relationships among each pair of individuals. For two individuals, *i* and *j*, covariance (*C*) was calculated as

$$C_{\mathrm{ij}}=\frac{1}{m}\sum_{s=1}^{m}\frac{\left(g_{\mathrm{is}}-x_{\mathrm{ij}}p_{s})(g_{\mathrm{js}}-x_{\mathrm{ij}}p_{s}\right)}{x_{\mathrm{ij}}p_{s}(1-p_{s})},$$

where *m* is the number of variable sites, *gis* is the genotype of individual *i* in site *s*, *x* is the average ploidy level of the two individuals, and *p* is the alternate allele frequency. We then conducted PCA on the matrix using the R function prcomp and extracted the first two axes of the rotated data for plotting. Changes in effective population size (*Ne*) over time were estimated in SMC++ (33) using all genotyped sites (both variant and invariant: 1,285,552 sites) filtered for quality, depth, and max 20% missingness as input. by specifying time points from 10 to 1,000,000 generations, mutation rate as 7e−9 (76), and a 1-y of generation time.

### Sequence Processing and Allele Frequency Estimation.

Likelihoods for the three possible genotypes in each biallelic site were then calculated from the BAM files in ANGSD 0.939 (77). Nucleotides with base qualities lower than 20 and reads with mapping quality lower than 30 were discarded. Sites with data missing from 20% or more of the 90 individuals were also excluded. Analysis of genetic diversity and population structure was performed in ANGSD using the empirical Bayes method to calculate Tajima’s D, which approximates how far the population is from a mutation-drift balance (78). SplitsTree (79) was used to clarify the extent of gene flow between populations.

### Selection Scans.

Allele frequencies for the selection scan were estimated directly from the site allele frequency likelihoods based on individual genotype likelihoods assuming HWE (80) in ANGSD. Fst was estimated using Hudson’s estimator as defined in ref. 81, since it is independent of sample sizes even when Fst is not identical across populations. Selected sites were detected by scanning the contigs in 1 kb nonoverlapping windows for areas of localized extreme differentiation between the coastal and inland populations, which is a pattern indicative of directional selection (82). The regions with the highest 1% values were identified and considered as candidate regions under coastal selection. Fst distributions were plotted with ggplot2 package (83) using 1 kb nonoverlapping (step size 1,000 bp) sliding windows. PCAdapt was run with default parameters on the GATK-called, best practices filtered VCF (48).

### Transcriptome Analysis.

RNA from leaves and roots of ST and SS *B. fruticulosa* plants hydroponically cultivated under 0 mM or 150 mM NaCl was extracted using the Maxwell plant RNA kit (Promega Corporation, Madison, WI, USA) following the manufacturer’s instructions. Total RNA was quantified using a Qubit 2.0 Q32866 (Life Technologies, Carlsbad, CA, USA) and then used to prepare the complementary DNA (cDNA) library. cDNA library was composed by 48 samples (2 tissues × 2 treatments × 2 origins × 2 salinity phenotypes × 3 replications) and sequenced at the Illumina NovaSeq 6000 using standard procedures to generate 150 bp paired-end reads (Novogene, Sacramento, CA, USA). Quality assessment of raw sequencing data was performed with FastQC software. Two samples (one “Salt SS” and one “Control ST”) were excluded due to low RIN and low-quality scores (<Q10). Low-quality reads and sequencing adapters from raw data were removed with Trim Galore! software (https://github.com/FelixKrueger/TrimGalore)*. Rsubread* package was used to map the paired-end reads to the reference genome (84) and *samtools* (85) was used for sorting and indexing. HTSeq-counts were applied to count the overlap of reads with the gene models (86).

PCA of root and leaf data was performed on the whole transcript levels. The differential expression analysis was performed with DESeq2 R Bioconductor package (1.39.2) (87). DEGs were selected based on L2FC > |2|, *P*-adj < 0.05.

### Enrichment Analysis.

GO terms were obtained with a customized “*B. fruticulosa* GO univers” created with orthofinder (88) using orthologous genes of *A. thaliana* and *R. sativus*. Fisher’s Exact Test was used to determine enrichment using *elim* algorithm in topGO packages of Bioconductor. STRING platform was used to visualize protein–protein interaction networks and perform enrichment analysis with the genes with *A. thaliana* orthologues. The TF with significantly overrepresented target number and the regulations between the TFs and the input genes were obtained using the “TF Enrichment” online tool of the Plant Transcriptional Regulatory Map (https://plantregmap.gao-lab.org/tf_enrichment.php).

### EAA.

To identify candidate genes associated with particular soil ionome characteristics, we performed EAA using LFMMs—LFMM 2 [https://bcm-uga.github.io/lfmm/; (47)] using the soil and leaf ionome as phenotype. We tested the association of allele frequencies at each SNP for each individual with associated soil concentration of elements differentiating saline and nonsaline soils. We retained 1,080,632 SNPs without missing data and MAF > 0.05 as an input for the LFMM analysis. LFMM accounts for a discrete number of ancestral population groups as latent factors. We used three latent factors reflecting the number of genetics clusters in our dataset. To identify SNPs significantly associated with soil variables, we have used a *P*-value threshold of 1 × 10^−5^. Finally, we annotated the candidate SNPs to genes, termed “LFMM candidates” (at least one significantly associated SNP per candidate gene).

### Climatic Data.

Monthly precipitation (mm), reference evapotranspiration (ET_0_, mm), and maximum, minimum, and mean temperature (°C) from 2010 to 2023 were obtained from the “Servei Metereologic de Catalunya” (https://ruralcat.gencat.cat/web/guest/agrometeo) selecting Roses and Cabanes stations for the North region and Malgrat de Mar and Viladrau stations for the Central region. ET_0_, or theoretical evapotranspiration in a soil covered with a uniform 10 cm high turf cover, was calculated according to the Penman-Monteith methodology (89).

### Statistical Analysis.

One‐way or multivariate ANOVA (ANOVA/MANOVA) was used to test for significant differences (*P* < 0.05) between means of data with respect to growth, physiological parameters, ionome, fitness, gene expression, and climatic data. To test for correlations between two variables, a bivariate fit was applied. To perform multiple comparisons of group means, Tukey's HSD test was conducted. A permutation test was performed to determine whether the number of overlaps between DEGs or GO categories observed was likely to occur by chance. For each condition (DEGs, Fst, EAA, PCAdapt) a list of random genes the same size as the real candidate gene list was generated using all the genes in the genome. GO analyses were then performed on these random lists and the number of overlapping GO categories was determined. This was performed 10,000 times for both the North and Central candidate gene lists. *P*-values were calculated as X/10,000 where X is the number of times there was an equal or greater number of overlapping GO terms in the randomized test vs. the real data (Datasets S6 and S11). Statistical analyses and plots were performed using SAS Software JMP (v.16.0) or R (version 4.2).

## Supplementary Material

Appendix 01 (PDF)

Dataset S01 (XLSX)

Dataset S02 (XLSX)

Dataset S03 (XLSX)

Dataset S04 (XLSX)

Dataset S05 (XLSX)

Dataset S06 (XLSX)

Dataset S07 (XLSX)

Dataset S08 (XLSX)

Dataset S09 (XLSX)

Dataset S10 (XLSX)

Dataset S11 (XLSX)

Dataset S12 (XLSX)

## Data Availability

PromethION, Illumina sequencing, genome assembly and RNAseq raw data generated in this study have been deposited in the European Nucleotide Archive under the project accession PRJEB74663 (90).
