# Supplementary material for: Local cryptic diversity in salinity adaptation mechanisms in the wild outcrossing Brassica fruticulosa
Source: Proc Natl Acad Sci U S A. 2024 Sep 24;121(40):e2407821121. doi: 10.1073/pnas.2407821121 (PMC11459175; doi:10.1073/pnas.2407821121)
Supplement: Supplementary file 1 — Appendix 01 (PDF) [file pnas.2407821121.sapp.pdf]

Supporting Information for

Local cryptic diversity in salinity adaptation mechanisms in the wild outcrossing *Brassica fruticulosa*

Silvia Busoms[1]*, Ana C. da Silva[2,3], Glòria Escolà[1], Raziyeh Abdilzadeh[2], Emma Curran[2], Anita Bollmann-Giolai[4,5], Sian Bray[2], Michael Wilson[6], Charlotte Poschenrieder[1] and Levi Yant[2,7,]*

[1] Department of Plant Physiology, Universitat Autònoma de Barcelona, 08193 Barcelona, Spain
[2] School of Life Sciences, University of Nottingham, Nottingham, NG7 2RD, United Kingdom
[3] Present address: Department of Veterinary Medicine, University of Cambridge, UK
[4] Department of Cell and Developmental Biology, John Innes Centre, Norwich Research Park, NR4 7UH, Norwich, UK
[5] Present address: Department of Molecular Biology, Max Planck Institute for Biology, Tübingen, Germany
[6] School of Biosciences, University of Nottingham, Nottingham, NG7 2RD, United Kingdom
[7] Department of Botany, Faculty of Science, Charles University, Prague, Czech Republic

Silvia Busoms and Levi Yant
Email: silvia.busoms@uab.cat and levi.yant@nottingham.ac.uk

This PDF file includes:

Figures S1 to S6

Other supporting materials for this manuscript include the following:

Datasets S1 to S12

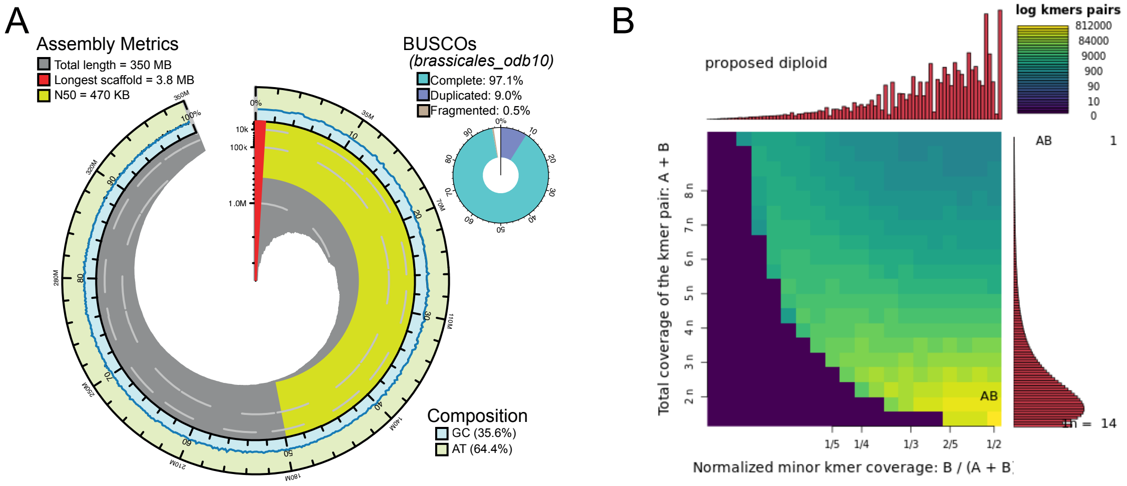

Figure S1. Long-read genome assembly of *B. fruticulosa* and ploidy confirmation of reference individual. (A) Snail plot with genome assembly and gene content metrics. **(B)** Smudgeplot ploidy confirmation of reference individual

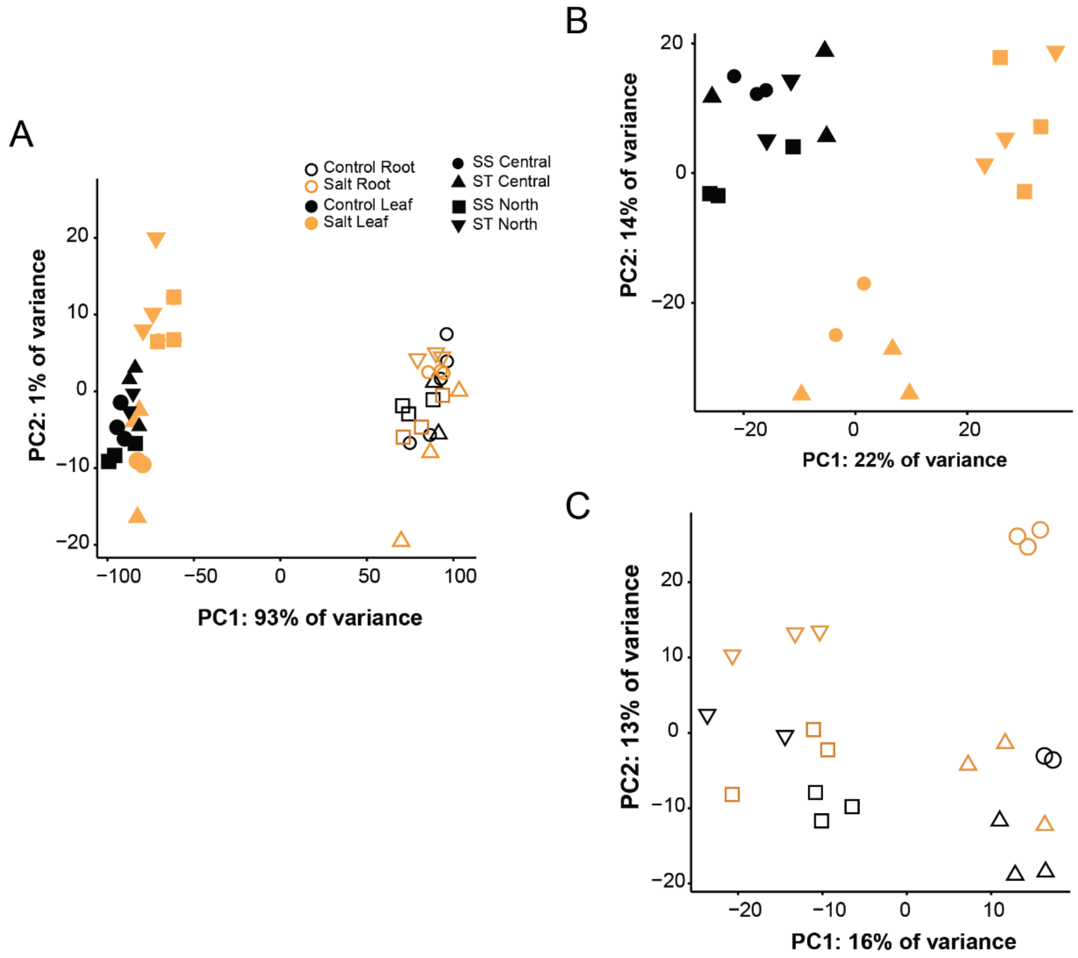

Figure S2. PCA of **(A)** all *Brassica fruticulosa* transcripts, **(B)** of leaf transcriptome profiles, and **(C)** root transcriptome profiles of 12 salt-tolerant (triangles up/down) and 12 salt-sensitives (circle/square) *B. fruticulosa* individuals from north and central metapopulations treated with 0 mM NaCl (control, black) or 150 mM NaCl (salt, orange) for 10 days.

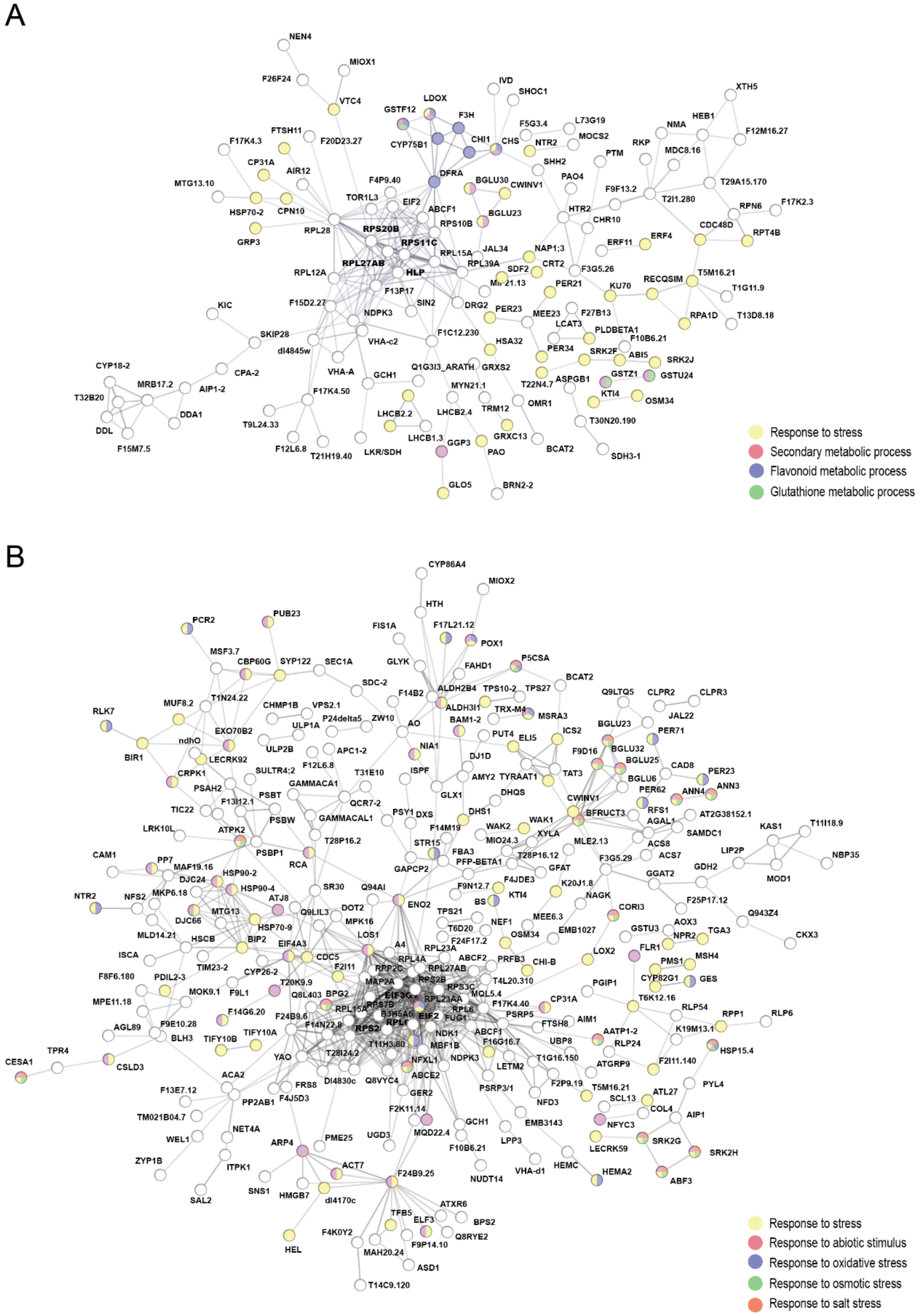

Figure S3. STRING networks of DEGs from the pairwise comparations of salt tolerant *vs* salt sensitive populations under salt stress of **(A)** north populations **(B)** central populations. Each sphere corresponds to one gene and nodes represent protein-protein interactions Only connected proteins are represented and proteins from relevant enriched GO terms are coloured.

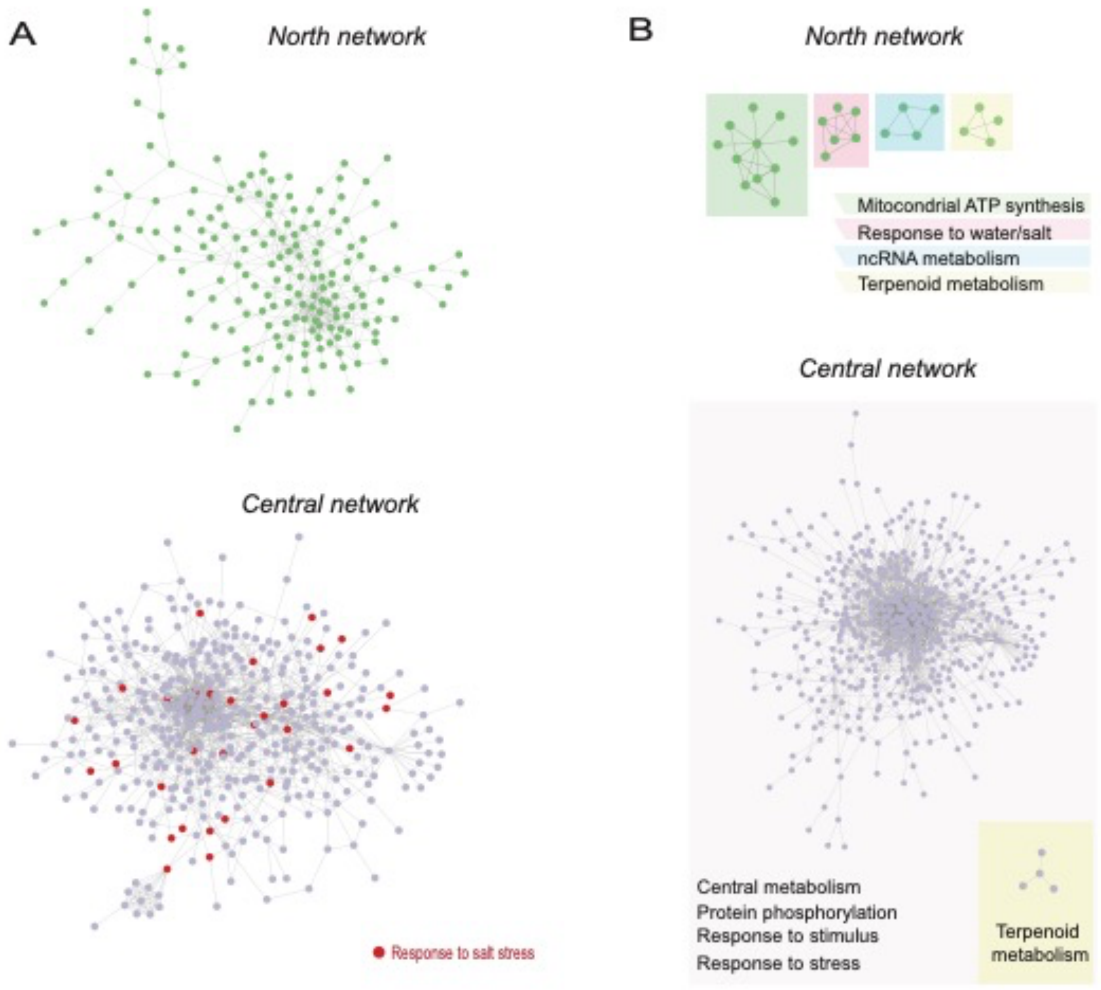

Figure S4. (**A**) Probabilistic functional gene networks of DEGs from the central comparison (purple circles) and from the north (green circles). ´Response to salt stress' subcommunity is indicated with red circles. (**B**) Probabilistic functional gene networks of Coastal Selection Candidates (Fst 1% outliers) from the North (green circles) and the Central (purple circles) clusters. Subcommunities are annotated according to significant enrichment in Gene Ontology terms for biological processes (*adj-pval* < 0.05, Fisher's exact test).

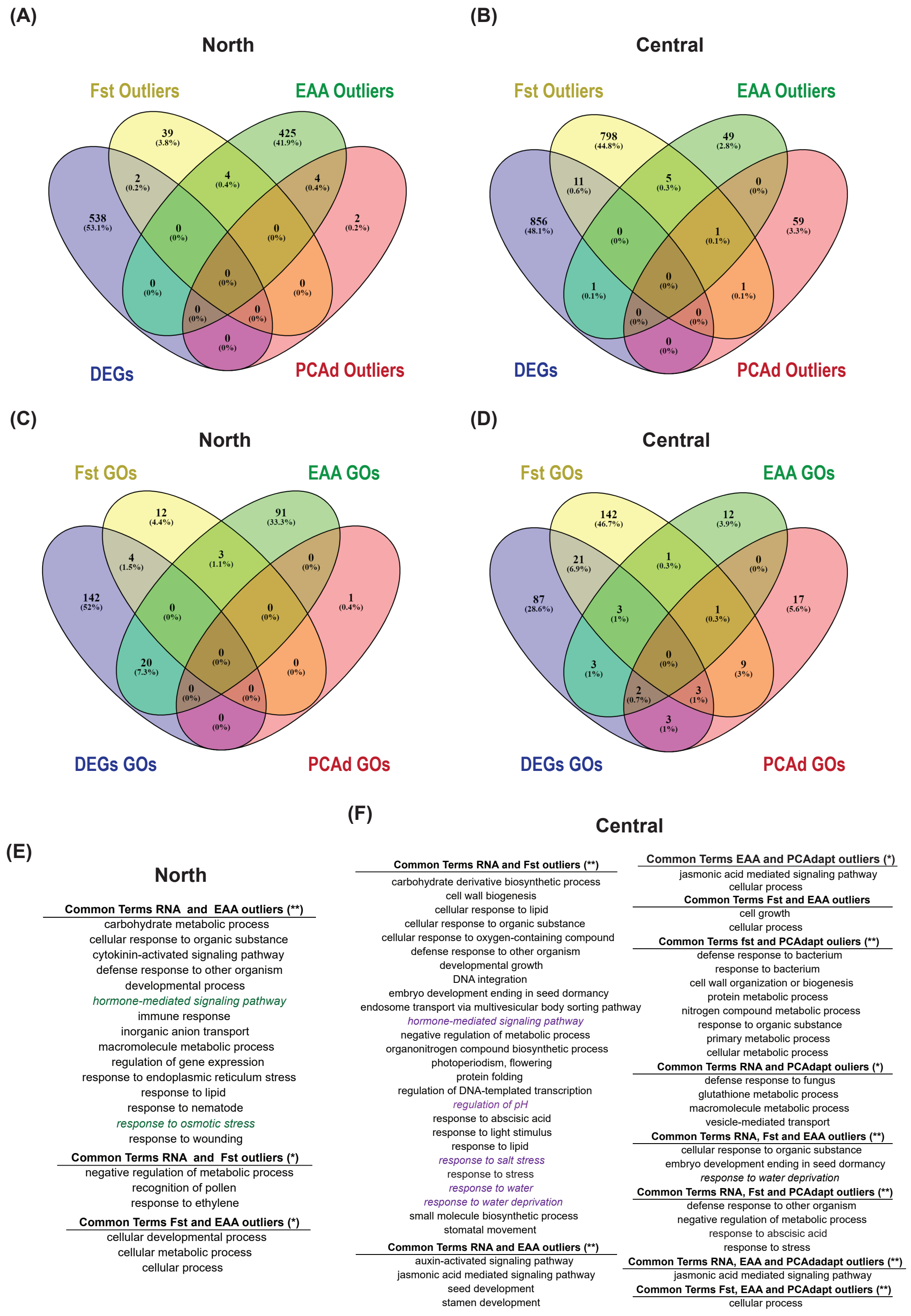

Figure S5. Candidate gene overlaps of *B. fruticulosa* north (**A**) and central (**B**) genomic (Fst, EAA, and PCAdapt outliers) and transcriptomic (DEGs) analysis. Overlap of the enriched GO terms of the candidate genes from north (**C, E**) and central (**D, F**) genomic and transcriptomic analysis. Asterisks indicate significant contrasts (Permutation test; * < 0.05 threshold, ** < 0.01 threshold).

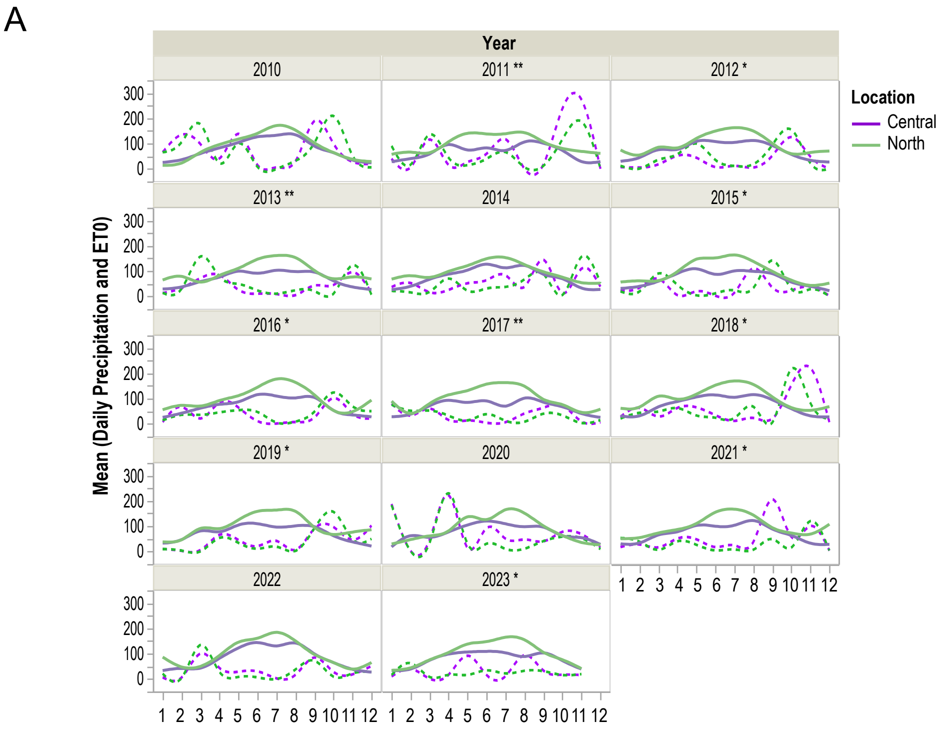

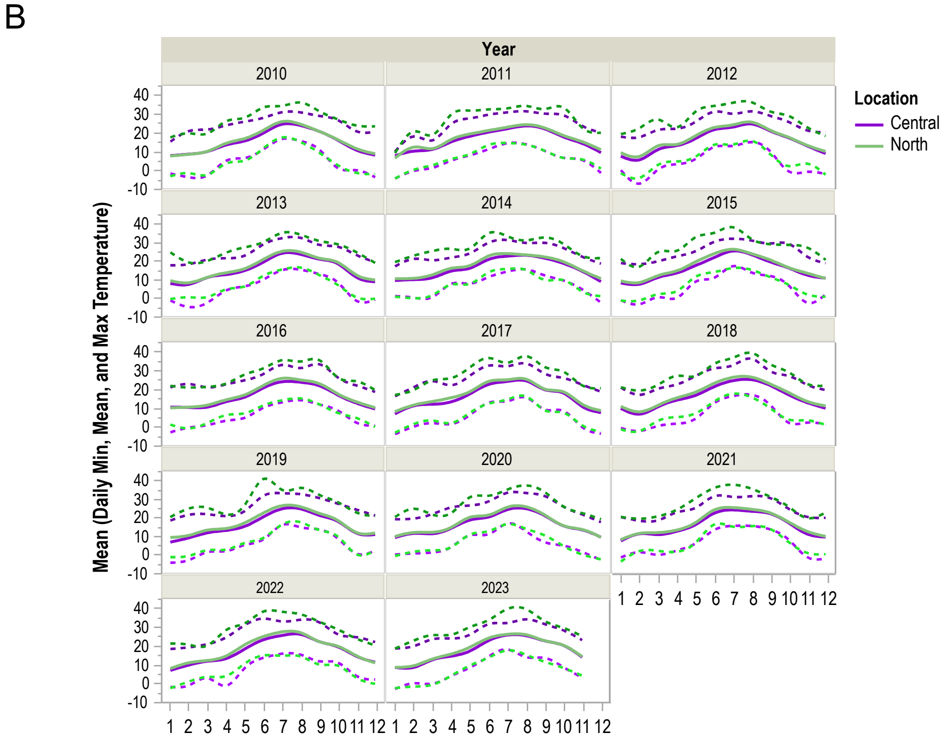

Figure S6. (A) Monthly mean precipitation and **(B)** monthly mean minimum, mean and maximum daily temperature of 14 years (2010-2023) recorded in Roses (north, green line) and in Malgrat de Mar (central, purple line) stations.
